# Editorial: Molecular mechanisms of bacterial disease in cultured fishes

**DOI:** 10.3389/fvets.2022.1073631

**Published:** 2022-11-17

**Authors:** Musa Najiah, Lixing Huang, Huanying Pang, Valentina Virginia Ebani

**Affiliations:** ^1^Faculty of Fisheries and Food Science, Universiti Malaysia Terengganu, Kuala Nerus, Malaysia; ^2^Fisheries College, Fujian Engineering Research Center of Aquatic Breeding and Healthy Aquaculture, Jimei University, Xiamen, China; ^3^Fisheries College, Guangdong Ocean University, Zhanjiang, China; ^4^Department of Veterinary Sciences, University of Pisa, Pisa, Italy

**Keywords:** bacterial infection, bacterial communication, bacteria-host interaction, virulence regulation, virulence factors interaction

Aquaculture is the fastest growing food production industry in the world, contributing nearly half of the global fish consumption. The world aquaculture recorded a total production (land and marine) of 87.5 million tons in 2020, which is a huge leap forward from 21.8 million tons in the 1990s (1). Aquaculture is facing diseases of various etiologies including bacteria. Although antimicrobial treatment is permitted under strict regulation, antimicrobial use should be refrained from in light of the risk of inducing antimicrobial resistance. It is crucial to seek novel effective solutions toward a healthier aquaculture practice, which requires us to have a clearer and deeper understanding of the disease mechanisms, including the host responses. Bacterial disease mechanisms are complicated, and vary considerably between bacteria. This Research Topic presents the current state of knowledge in molecular mechanisms of bacterial diseases in cultured fishes.

## Bacterial communication and regulatory mechanism

Bacteria use quorum sensing (QS) to communicate with one another via signal molecules called autoinducers (AIs), and coordinate their behaviors for survival according to cell density. This subsequently activates QS transcriptional regulator to control functions such as biofilm formation, motility, bioluminescence, secretion, and virulence. These dynamically interconnected communication and regulatory mechanisms remain to be fully elucidated. Bacteria use different QS-dependent strategies to compete for space, including clustered regularly interspaced short palindromic repeats (CRISPR) and type VI secretion systems (T6SSs). While T6SSs function primarily in competition against rival bacteria in polymicrobial environments, they also involve in pathogenesis of some diseases. In the study of QS and survival strategies of *Aliivibrio wodanis* that co-exists with *Moritella viscosa* in winter ulcer disease in Atlantic salmon, Maharajan et al. demonstrate that cell density and temperature influence QS-related genes, where low temperature (at which winter ulcer occurs) activates AHL-mediated AinS/AinR system that regulates CRISPR-Cas and T6SSs via LitR master QS regulator. The interactions between *A. wodanis* and *M. viscosa*, and with the host is, however, yet to be investigated.

## Bacteria-parasite interaction

While bacterial co-occurrence in disease such as that of winter ulcer disease needs explanation, bacteria-parasite interaction in the form of co-infection is also a concern in aquaculture. Dinh-Hung et al. describe co-infection of myxozoan gill parasite, *Henneguya* sp. with a novel intracellular Chlamydia-like organism, *Candidatus Piscichlamydia trichopodus* in snakeskin gourami *Trichopodus pectoralis*. Further studies on how parasites and bacteria co-interact with host may provide insight into the molecular mechanisms of disease. Could there be similarity with the probable remote “talk” between gill monogenean, *Dactylogyrus lamellatus* and host intestinal microbiota (2)?

## Bacterial persistence

Bacterial persistence is a phenomenon where very small subpopulations of isogenic bacterial cells (persister cells) undergo dormant cell cycle to survive sudden environmental insults such as antibiotic treatments. The dormancy state confers high tolerance to multiple antibiotics, but the mechanisms involved are still not identified. Here Jiang et al. report the use of selected amino acids and saccharides to revert *Vibrio splendidus* persister cells to allow complete elimination by tetracycline treatment.

## Host immune response to bacterial infection

The immune response mechanisms against bacterial infection are behind the host tolerance and resistance to bacterial diseases. In the transcriptomic analysis of *Vibrio harveyi* infection in *Takifugu rubripes*, Gao et al. show that nucleotide-binding and oligomerization domain (NOD)-like receptor signaling pathway and cytokine-cytokine interaction are most enriched in spleen and gill, respectively. This study provides an understanding into immune response mechanisms and development of disease resistance markers against *V. harveyi*.

## Mobile genetic elements

Virulence associated mobile genetic elements are widespread in the aquatic environment among various genera of bacteria e.g., *Vibrio, Photobacterium damselae* and *Staphylococcus aureus*. There are also evidences that *Aeromonas* can actively involve in transferring genetic material with phylogenetically distant bacteria. *Aeromonas* pathogenicity has been attributed to multiple virulence factors contributing to different mechanisms of infection, including haemolysins, proteases, elastases, lipases, enterotoxins, phospholipases, chitinases, and deoxyribonucleases. Of these, pore-forming toxins (encoded by *aer*A gene) and cytotoxic enterotoxins (by *act, alt*, and *ast* genes) are the primary elements of virulence in *Aeromonas*. In this Research Topic, Mangar et al. examine the virulence factors of *A. veronii*, as well as severity of lesions and mortality induced in *Anabas testudineus* in association with *aer/haem, ascV, fla*, and *aspA*.

## T6SS transcriptional activator

In a study of virulent *A. hydrophila* from grass carp *Ctenopharyngodon idella*, Li et al. reveal that σ54-transcriptional activator, VasH is strictly required for T6SS functionality. This supports that VasH protein not only contributes to bacterial cytotoxicity, anti-host killing (resistance against host immune cleaning), but is also needed for systemic dissemination of *A. hydrophila*.

## Induction of host cell apoptosis

Many bacteria are able to induce apoptosis in infected host cells to gain access to tissues. Alanine dehydrogenase (ALD) is a microbial enzyme catalyzing interconversion between alanine and pyruvate in microbial metabolism. Chen et al. describe an ALD homolog (NsALD) from *Nocardia seriolae* that causes chronic, systemic, granulomatous disease in aquaculture. They show that NsALD activates caspase-3 and triggers apoptosis in host cells.

Besides the molecular aspects highlighted in the seven articles above, this Research Topic also incorporates one article each on antimicrobial resistance (AMR), and advancement in disease detection. AMR is a global threat to animal and human health. Antimicrobial residues resulting from aquaculture use not only pose a food safety concern, but also trigger emergence of multi-antimicrobial resistance in bacteria including those in the wild species (3). Liao et al. investigate the AMR profile and associated genes in *Escherichia coli* from aquaculture farms (water, soil, sediment). They reveal the resistance rates to 23 antimicrobials, and the detection rates of AMR genes. Acute hepatopancreatic necrosis disease (AHPND) is a disease that can cause high mortality in cultured penaeid shrimp. AHPND is mainly caused by *Vibrio* species bearing pVA1 plasmid that encodes pirA_VP_ and pirB_VP_ toxins. Li et al. describe the development of recombinase polymerase amplification (RPA)-CRISPR/Cas12a assay for detection of *pirA*_*VP*_ and *pirB*_*VP*_ genes, and coupled with lateral flow strip readout.

## Concluding remarks

The molecular mechanisms of bacterial diseases in cultured fishes are yet to be fully understood. The bacteria-host and virulence factors interaction much remain to be elucidated. This Research Topic lays a foundation for further investigation into the mechanisms involved, and provides new insights into molecular basis of pathogenicity. A better understanding at molecular level will allow for effective risk monitoring for bacterial diseases.

## Author contributions

All authors listed have made a substantial, direct, and intellectual contribution to the work and approved it for publication.

## Conflict of interest

The authors declare that the research was conducted in the absence of any commercial or financial relationships that could be construed as a potential conflict of interest.

## Publisher's note

All claims expressed in this article are solely those of the authors and do not necessarily represent those of their affiliated organizations, or those of the publisher, the editors and the reviewers. Any product that may be evaluated in this article, or claim that may be made by its manufacturer, is not guaranteed or endorsed by the publisher.

## References

[1] FAO. The State of World Fisheries and Aquaculture 2022. Towards Blue Transformation. Rome: FAO (2022). p. 3. 10.4060/cc0461en

[2] Wang L Zhang D Xie J Chang O Wang Q Shi C. Do ectoparasites on fish gills “talk” with gut microbiota far away? Aquaculture. (2023) 562:738880. 10.1016/j.aquaculture.2022.738880

[3] Najiah M Nadirah M Sakri I Shaharom-Harrison F. Bacteria associated with wild mud crab (*Scylla serrata*) from Setiu Wetland, Malaysia with emphasis on antibiotic resistances. Pak J Biol Sci. (2010) 13:293–7. 10.3923/pjbs.2010.293.297 20506717

